# Antiplatelet Treatment Strategy in MINOCA Patients: Predictors of Decision Making in Clinical Practice and Prognostic Implications

**DOI:** 10.3390/jcm14113984

**Published:** 2025-06-05

**Authors:** Emmanouil Mantzouranis, Ioannis Leontsinis, Panayotis K. Vlachakis, Constantinos Mihas, Panagiotis Iliakis, Eirini Dri, Athanasios Sakalidis, Stergios Soulaidopoulos, Christos Fragoulis, Anastasios Milkas, Eleftherios Tsiamis, Dimitrios Tsiachris, Kyriakos Dimitriadis, Konstantinos Tsioufis

**Affiliations:** 1 First Department of Cardiology, Hippokration Hospital, Athens Medical School, National and Kapodistrian University of Athens, Vas. Sofias 114, 11527 Athens, Greece; acardioekpa@gmail.com (I.L.); vlachakispanag@gmail.com (P.K.V.); gas521@yahoo.co.uk (C.M.); panayiotisiliakis@gmail.com (P.I.); drieirini@gmail.com (E.D.); asakalidis@gmail.com (A.S.); soulaidopoulos@hotmail.com (S.S.); christosfragoulis@yahoo.com (C.F.); ltsiamis@otenet.gr (E.T.); tsiacmed@yahoo.com (D.T.); dimitriadiskyr@yahoo.gr (K.D.); ktsioufis@gmail.com (K.T.); 2 Athens Naval Hospital, 11521 Athens, Greece; tmhlkas@otenet.gr

**Keywords:** MINOCA, Takotsubo syndrome, antiplatelet therapy, coronary artery disease, cardiac magnetic resonance

## Abstract

**Background/Objectives:** Large clinical trials have established the optimal antiplatelet strategy in the wide spectrum of coronary artery disease. However, data are scarce regarding MINOCA and the aim of our study is to present data from the current clinical practice. **Methods**: A total of 151 patients were included in this study after exclusion of 27 patients with myocarditis and other diagnoses. A cardiac magnetic resonance (CMR) performed at 123/151 patients demonstrated an ischemic pattern of late gadolinium enhancement (LGE) confirming the diagnosis of true acute myocardial infarction (AMI) in 42 cases (28%). Based on multimodality imaging and clinical judgement, Takotsubo syndrome (TTS) was diagnosed in 55 patients (36%), whereas CMR failed to reveal abnormal findings in 54 cases (36%), categorized as MINOCA of unknown origin. **Results**: Regarding antithrombotic prescriptions at discharge, 38% of patients received dual antiplatelet (DAPT) or dual antithrombotic therapy (DAT, 1 antiplatelet plus 1 anticoagulant), 49.7% received single antiplatelet (SAPT) or anticoagulant, and 12% received no antithrombotic treatment. Univariate analysis showed that the likelihood of prescribing DAPT or DAT was associated with left ventricular ejection fraction (LVEF) (r = 0.202, *p* = 0.013), atherosclerotic lesions on coronary angiography (r = 0.303, *p* < 0.001), prior use of anticoagulants (r = −0.258, *p* = 0.001), and marginally with the INTERTAK score (r = −0.198, *p* = 0.044). A multivariable model, adjusted for age, LVEF, ECG abnormalities, and history of anticoagulant use, confirmed the independent association between angiographic evidence of atherosclerosis and the decision for DAPT/DAT (OR: 0.334, 95% CI: 0.307–0.813, *p* < 0.001). However, the initial treatment decision did not seem to impact 2-year prognosis in our population. **Conclusions**: Our study results reveal that decision making in the antithrombotic strategy for MINOCA patients poses a challenge in clinical practice. More robust data are required for definite conclusions on the prognostic implications.

## 1. Introduction

Myocardial infarction with non-obstructive coronary arteries (MINOCA) accounts for approximately 5–10% of all acute myocardial infarction (AMI) cases [1,2]. In this enigmatic clinical entity, the patient presents with a clinical picture fulfilling the diagnostic criteria for AMI, but coronary angiography does not reveal any significant stenosis (defined as ≥50%) and no other obvious diagnosis is identified, thus posing a diagnostic and therapeutic challenge. The pathophysiological substrate is highly heterogeneous, including plaque disruption, coronary vasospasm, microvascular dysfunction, and spontaneous coronary artery dissection (SCAD), each with distinct implications for management and prognosis [3,4]. On the other hand, differential diagnosis from Takotsubo syndrome (TTS) and myocarditis can be really challenging and the performance of cardiac magnetic resonance (CMR) is warranted in most cases [5].

In contrast to obstructive acute coronary syndromes (ACSs), where dual antiplatelet therapy (DAPT) forms the cornerstone of secondary prevention, there is a notable absence of guideline-directed recommendations for antiplatelet use in MINOCA [3,6]. While large randomized clinical trials (RCT) have established the benefit of DAPT in obstructive ACS [7,8,9] and a wide spectrum of coronary artery disease (CAD) [10], the evidence is scarce in MINOCA as it remains limited to retrospective analyses, observational cohorts, and post hoc registry data [11,12]. Notably, studies such as the SWEDEHEART registry have shown that DAPT use in MINOCA patients does not translate into an improvement of prognosis, raising concerns about the potential overtreatment of patients without confirmed thrombotic etiology. On the other hand, antiplatelet therapy has not shown any benefit in the setting of TTS [13,14].

The challenge of selecting an appropriate antiplatelet regimen in MINOCA is greatly attributed to the diagnostic uncertainty at the time of presentation. MINOCA at presentation is a working diagnosis and a thorough diagnostic approach is warranted to differentiate true AMI from mimicking conditions such as TTS or myocarditis. CMR is of critical importance in these cases [6,15], often leading to a reconsideration of initial diagnosis and modification of the treatment strategy [5,16,17]. Moreover, the increasing utilization of intracoronary imaging modalities, such as optical coherence tomography (OCT) and intravascular ultrasound (IVUS) are enhancing our ability to identify mechanisms such as plaque erosion, and when combined with CMR, greatly increase our diagnostic yield [18].

In this context, our study aimed to explore the current clinical practice of antiplatelet treatment in patients with a working diagnosis of MINOCA, identify predictors influencing treatment decisions, and assess the long-term prognostic implication of these decisions.

## 2. Materials and Methods

### 2.1. Study Aim and Design

The present study is a single center prospective observational cohort involving patients aged 35 to 85 years old admitted to the cardiology ward or the coronary care unit (CCU) of a tertiary hospital, fulfilling the criteria for the working diagnosis of MINOCA, excluding patients diagnosed with myocarditis or cardiomyopathy. The population was further divided into patients diagnosed with MINOCA or Takotsubo syndrome based on the results of multimodality imaging and complete clinical assessment. Patients were followed up for 24 months in order to record data of contemporary clinical practice regarding the management of these conditions and its implications in terms of prognosis, with emphasis on antithrombotic treatment strategies.

### 2.2. Study Population

This study included all patients aged 35 to 85 years who were admitted to the cardiology clinic or the CCU of the Hippokration General Hospital of Athens and fulfilled the criteria for the working diagnosis of MINOCA between 1 October 2019, and 31 October 2022. Patients with other prevalent diagnoses emerging during hospitalization were excluded. All remaining patients underwent an invasive coronary angiogram and were treated according to European guidelines and local hospital protocols. The final diagnosis was documented by CMR with an effort to be performed within 30 days, provided there were no findings consistent with myocarditis or cardiomyopathy. In cases where CMR could not be performed, the final diagnosis and decision regarding patient inclusion were made by a medical council based on multimodality imaging data and comprehensive clinical assessment.

Subsequently, the population was categorized based on the final diagnosis as either MINOCA or Takotsubo syndrome. Patients with MINOCA were further classified into true AMI based on the presence of an ischemic pattern on the late gadolinium enhancement (LGE) sequence and MINOCA of unknown origin if the CMR was non-pathological or not performed.

All participants were informed about the aim of this study and subsequently signed an informed consent form to participate.

### 2.3. Ethics

This study has been approved by the Ethical Committee of the Hippokration General Hospital of Athens and by the Medical School of the National and Kapodistrian University of Athens. All participants or legal representatives provided written informed consent. This study was conducted in accordance with the ethical principles of the Declaration of Helsinki, and all patient data were collected and stored following the local GDPR legislations.

### 2.4. Study Protocol

#### 2.4.1. Baseline Data Collection

Patient data were collected using a specially designed, printed, and electronic case report form (CRF). These data included demographic characteristics, past medical history, and laboratory and hemodynamic parameters. Vital signs upon arrival at the emergency department were also documented, as well as data from electrocardiography, echocardiography, and coronary angiography. During hospitalization, patients underwent laboratory testing for the evaluation of renal function (eGFR using Cockcroft-Gault, MDRD formulas), metabolic profile, liver function, hematological parameters, serial measurements of myocardial necrosis markers, natriuretic peptides and inflammation markers (CRP). INTERTAK score was calculated as described in the literature by adding up points for each of the seven prespecified parameters as follows: female sex, 25 points; emotional trigger, 24 points; physical trigger, 13 points; absence of ST—segment depression, 12 points; psychiatric disorders, 11 points; neurologic disorders, 9 points; and QTc prolongation, 6 points. This score was used both as a clinical tool and as a continuous parameter [19].

Medication at admission, during hospitalization, and at discharge was thoroughly recorded. Regarding the prescription of antiplatelet therapy, the following categories were defined:

- No antithrombotic therapy.
- Single antiplatelet therapy (SAPT) or oral anticoagulants (OACs): use of single antiplatelet therapy (aspirin) or ONLY an anticoagulant (usually in the context of atrial fibrillation).
- Dual antiplatelet therapy (DAPT), or a combination of one antiplatelet with one anticoagulant—dual antithrombotic therapy (DAT), or a combination of two antiplatelets with one anticoagulant—triple antithrombotic therapy (TAT). In the present study, there were no patients receiving TAT.

#### 2.4.2. Follow-Up Period

Patients were evaluated at 1 month with an in-person visit, during which the results of the CMR were reviewed to establish the final diagnosis. Follow-up was conducted via telephone at 3 and 6 months to assess for the possible occurrence of major adverse cardiovascular events (MACEs). In the case of a reported event, an in-person clinic visit was scheduled for the patient to provide the relevant medical documentation for validation of the event and to reassess their clinical status and medical therapy. Therapeutic decisions were not made by the study investigators.

At 12 and 24 months, follow-up clinic visits were again scheduled to assess primary outcomes. In the case of reported events, the corresponding medical documents were collected for validation.

#### 2.4.3. Endpoint Definition

The following major cardiovascular outcomes (MACE) were assessed during the follow-up period:

- Hospitalization for new ACS (NSTEMI/STEMI/Unstable Angina) or need for revascularization.
- Hospitalization for heart failure.
- Hospitalization for stroke (confirmed by imaging).
- Hospitalization for clinically significant arrhythmia (including atrial fibrillation, ventricular tachycardia/need for implantable defibrillator, or conduction disturbance requiring pacemaker implantation).
- Cardiovascular and all cause death.
- Composite outcome of cardiovascular morbidity and/or mortality.

The composite outcome was defined as the primary endpoint and each separate MACE was considered as a subsequent endpoint.

### 2.5. Statistics

Data analysis was performed using the statistical software IBM SPSS Statistics for Windows, Version 24.0 (IBM Corp., Armonk, NY, USA) Continuous variables with a normal distribution are presented as mean ± standard deviation, whereas those without a normal distribution are reported as median and IQR. Categorical variables are presented as frequencies and percentages. Comparisons between categorical variables were carried out using the chi-square (χ^2^) test. Comparisons of means for normally distributed continuous variables were performed using the unpaired Student’s *t*-test, while for non-normally distributed variables, the Mann–Whitney U test was used. Normality of distribution was assessed using the Kolmogorov–Smirnov test. To assess correlations between variables, either Pearson’s correlation coefficient, Phi coefficient, or Spearman’s rho was used, depending on the nature of the data. Differences in comparisons were considered statistically significant for *p*-values < 0.05. This was also valid in the assessment of the primary endpoint. However, in regards to the five different subsequent endpoints, the Bonferroni correction was employed, adjusting the significance threshold to *p* < 0.01 (0.05/5). Tests for associations were performed using linear regression for continuous dependent variables and binary logistic regression for binary variables. To evaluate the association between cardiovascular events and all-cause mortality with the parameters of interest, Kaplan–Meier survival curves were constructed and compared using the log-rank test. In addition, univariate and multivariate analyses were conducted using linear/logistic regression and Cox proportional hazards regression models.

## 3. Results

### 3.1. Patient Characteristics

A total of 183 patients were admitted initially fulfilling the diagnostic criteria of MINOCA (71% female; mean age: 62 ± 12 years old). Patients in whom an alternative prevalent diagnosis was identified were excluded from further analyses. In particular: three patients with type 2 myocardial infarction due to severe anemia and significant aortic valve stenosis, one patient with pulmonary embolism, and one patient with cholecystitis. Based on CMR findings, an additional 27 patients (14.7%) with typical findings of myocarditis were excluded [20,21].

**Figure 1:**
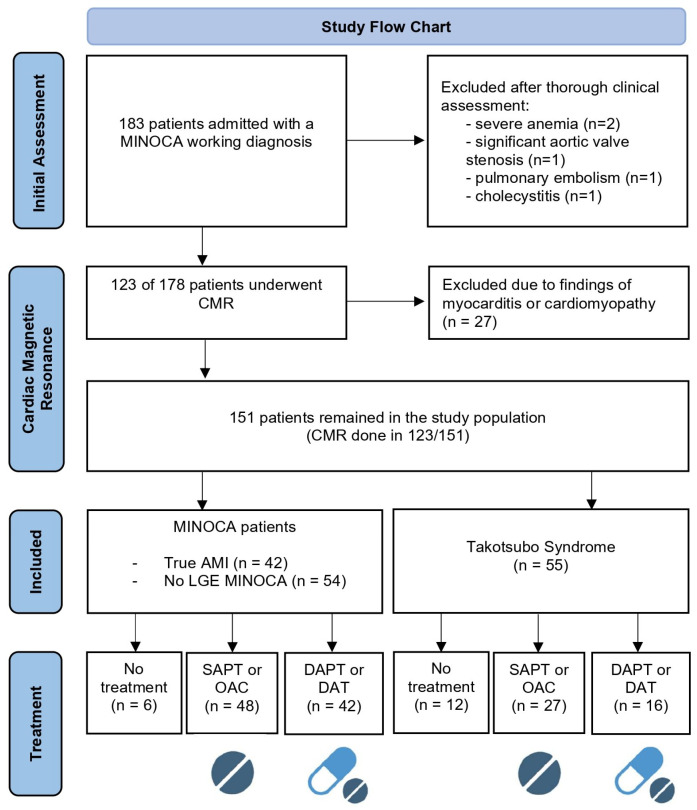
Study flow chart. AMI: acute myocardial infarction; CMR: cardiac magnetic resonance; SAPT: single antiplatelet therapy; DAPT: dual antiplatelet therapy; DAT: dual antithrombotic therapy (1 antiplatelet plus 1 anticoagulant); OAC: oral anticoagulant; LGE: late gadolinium enhancement. Based on PRISMA flow diagram 2020 [23] and created with biorender.org.

Of the 151 patients who remained in the study population, CMR was performed in 123 patients with a median time from admission of 19 days (IQR 1–144) and demonstrated an ischemic pattern of LGE, confirming the diagnosis of true AMI in 42 cases (28%). Based on multimodality imaging and clinical judgement [13,22], TTS was diagnosed in 55 patients (36%), whereas CMR failed to reveal abnormal findings or was deferred in 54 cases (36%) non-compatible with TTS diagnosis, who were categorized as MINOCA of unknown origin. Specifically, CMR was not performed in 14 patients with TTS and 14 patients with MINOCA (Figure 1).

Baseline demographics and clinical, laboratory, and imaging characteristics are presented in Table 1.

### 3.2. Antithrombotic Treatment Strategy

#### 3.2.1. Predictors for Decision Making

Regarding antiplatelet prescriptions of the total study population at discharge, 38% of patients received DAPT or DAT, 49.7% SAPT or anticoagulant, and 12% no antithrombotic treatment.

Among the patients who received a final diagnosis of MINOCA after CMR, 44% were discharged with a recommendation for DAPT or DAT, 50.5% with SAPT or only an anticoagulant, while 6% received neither antiplatelet nor anticoagulant therapy, presumably attributed to a high clinical suspicion of TTS or myocarditis.

On the other hand, among the patients with a final diagnosis of TTS, 29% were discharged with a recommendation for DAPT or DAT, 48% with SAPT or only anticoagulant, and only 7.8% received no antithrombotic therapy. It should be noted, though, that 23.6% of patients with TTS had atherosclerotic vessels with stenoses up to 50%.

Univariate analysis showed that the likelihood of prescribing DAPT or DAT versus SAPT/single anticoagulant or no treatment, was associated with LVEF (r = 0.202, *p* = 0.013), the presence of atherosclerotic lesions on coronary angiography (r = 0.303, *p* < 0.001), prior use of anticoagulants (r = −0.258, *p* = 0.001), and marginally with the INTERTAK score (r = −0.198, *p* = 0.044). On the contrary, no correlation was noticed with age, cardiovascular risk factors, renal function, ECG abnormalities, troponin levels, prior use of antiplatelet agents or hemoglobin value.

A multivariable binary logistic regression model, adjusted for age, LVEF, ECG abnormalities, and history of anticoagulant use, confirmed the independent association between angiographic evidence of atherosclerosis and the decision to initiate DAPT/DAT versus less intensified antithrombotic strategies (OR: 0.334, 95% CI: 0.307–0.813, *p* < 0.001). Of note, CMR led to a change in the initial diagnosis from TTS to MINOCA in seven patients (9%) and from MINOCA to TTS in nine patients (21%), altering treatment accordingly (Table 2).

#### 3.2.2. Prognostic Significance Analysis for Antithrombotic Treatment

Follow-up data were collected for 129 patients within 24 months, including 46 out of 55 from the TTS group and 83 out of 96 from the MINOCA group. Major adverse events occurred in 31 patients (24%) of the total population, specifically in 10 patients with TTS (22%) and 21 patients with MINOCA (25.3%).

The median time from discharge to the first cardiovascular event was 120 days (IQR 19–750). A total of four deaths were recorded, three of which were due to cardiovascular causes. The most frequent cardiovascular events were new ACSs (NSTEMI/UA), of which two led to revascularization. No STEMI cases occurred. Hospitalization for heart failure was warranted in four patients, while two patients were hospitalized for ventricular tachycardia—one of whom received an ICD—and one patient experienced ischemic stroke. No significant differences were observed in the incidence or type of cardiovascular events between the MINOCA and TTS groups.

**Figure 2:**
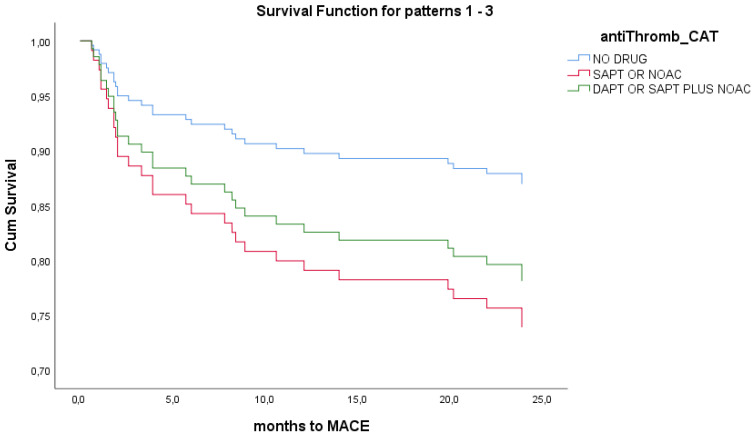
Univariate Cox-regression survival analysis for antithrombotic strategy in total population. No survival differences noted between different treatment groups at 24 months.

In order to assess the prognostic implications of the initial antithrombotic strategy, we performed a univariate Cox regression analysis for the total population of the study and separately for MINOCA and TTS groups. In the simple Cox regression analysis for the total population, there were no statistically significant differences in the composite primary endpoint of MACEs at 24 months among patients at the three different antithrombotic intensity classes (overall *p* = 0.546). Compared with patients not receiving antiplatelet therapy, the hazard ratios were 0.566 (95% CI 0.125–2.552, *p* = 0.458) for SAPT/OAC and 1.224 (95% CI 0.574–2.614, *p* = 0.601) for DAPT/DAT (Figure 2). Similar non-statistical findings were noted from separate analyses for MINOCA and Takotsubo. No multivariate analysis was performed. All *p*-values for the individual endpoints exceeded 0.05 and would remain non-significant after Bonferroni correction for multiple comparisons.

## 4. Discussion

Our study delves into the challenging landscape of antiplatelet therapy in patients with MINOCA, shedding light on the impact of clinical, imaging, and laboratory parameters in treatment decisions. Notably, our findings highlight the variability in clinical practice preference for SAPT and DAPT regimens in our patient cohort. Given the diverse underlying etiologies, ranging from plaque disruption to coronary artery spasm, the choice of antiplatelet therapy remains vague, demanding a nuanced understanding of individual patient presentations.

Moreover, our study underscores the significance of cardiac imaging, particularly CMR, in elucidating the underlying etiology in MINOCA patients. Reclassification of initial diagnosis is a common scenario, described in up to 70–80% of cases in the literature [5,13,14]. In our cohort, diagnosis was reconsidered in up to 21% of patients. The confirmation of true AMI through CMR findings emphasizes the importance of accurate diagnosis, which directly impacts the choice of antiplatelet therapy. In the Heart Attack Research Program-Imaging Study (HARP), a prospective study involving over 300 women diagnosed with MINOCA, investigators utilized multimodality imaging (CMR and OCT), and they revealed that in more than 50% of these cases, an ischemic pattern was identified [18]. Additionally, the utilization of CMR in approximately 23% of MINOCA individuals yielded inconclusive results, highlighting the necessity for additional research and suggesting the potential role of invasive coronary imaging to yield decisive insights in this “enigmatic” context [24].

In 2017, the SWEDEHEART national registry study examined 9466 MINOCA patients treated between 2003 and 2013, with 66.4% receiving DAPT. Notably, DAPT usage did not impact the composite endpoint of MACEs or lead to increased bleeding events; limitations include the absence of specific MINOCA etiologies and unreported details on P2Y12 inhibitor type and DAPT duration (11). In general, the existing information regarding the impact of antiplatelet usage in MINOCA individuals originates from registries or secondary scrutiny of RCTs and therefore lacks robustness. Nonetheless, the available data indicate that the employment of antiplatelet therapy, particularly DAPT, does not contribute to enhancements in overall clinical results [12]. However, it is imperative to consider that the utilization of CMR was not universal across all studies, and potential mimickers of MINOCA may have exerted an influence on the observational outcomes.

Notably, our results demonstrate the influence of abnormal angiographic findings on the decision to initiate DAPT, underlining the pivotal role of interventional imaging in guiding treatment strategies. As a result of this practice, 6% of patients ultimately diagnosed with MINOCA were discharged without any antiplatelet or anticoagulant therapy, while 29% of patients later confirmed to have TTS were initially discharged with a recommendation for DAPT or DAT. It should be emphasized, though, that 23.6% of patients with TTS had atherosclerotic vessels with stenoses up to 50%. Despite the aforementioned findings, the initial choice of antiplatelet strategy did not appear to impact long-term prognosis, either in the overall population or within the MINOCA and TTS subgroups. Prior use of anticoagulants was expected to impact decisions for antithrombotic intensity due to bleeding concerns, and according to our observations, the negative association shows that a patient already receiving a DOAC, usually for atrial fibrillation, was less likely to also receive an antiplatelet leading to DAT treatment.

In instances where there is evidence that MINOCA arises from plaque rupture/erosion, both the American Heart Association (AHA) and European Society of Cardiology (ESC) position statements advocate for the treatment of these patients similarly to those with conventional ACS, considering DAPT [2,3]. The definition of obstructive disease based on the angiographic 50% threshold is acknowledged as somewhat arbitrary yet pragmatic, aligning with prior AHA/ACC scientific statements [2]. However, given the subjective nature of this approach and the variability it introduces, it is imperative to recognize the potential limitations in accurately assessing lesion severity based solely on visual angiographic estimation, particularly considering the dynamic nature of lesions and the possibility of inter-angiogram variations. The use of physiological imaging techniques such as IVUS or OCT may play a critical role in refining the definition of MINOCA, providing a more comprehensive understanding of the underlying pathophysiological processes and the optimal treatment strategy in terms of intervention and medical treatment. Of note, the recently published PREVENT trial demonstrated that preventive percutaneous coronary intervention (PCI) of non-obstructive but vulnerable coronary plaques—defined as angiographically ≥50% but with fractional flow reserve (FFR) > 0.8 and assessed with intracoronary imaging—significantly reduced MACEs compared to optimal medical therapy alone [25]. In this line, the ongoing PROMISE trial is anticipated to provide additional insights into the influence of DAPT plus implantation of stent on the long-term prognosis of MINOCA patients with confirmed AMI of this nature [26]. Furthermore, novel emerging technologies have shown promising results in identification of plaque vulnerability and could potentially be applied in the near future in the context of MINOCA and ischemia with non-obstructive coronary arteries (INOCA) [27].

More novel approaches have considered the potential role of drug-coated balloons (DCBs) in the management of MINOCA with plaque disruption [28]. Also, the possible use of bioresorbable scaffolds (BRSs) seems intriguing in cases of plaque erosion or rupture considering the favorable plaque modification, preservation of endothelial function, and restoration of vessel physiology [29]. Given the heterogeneous nature of the underlying pathophysiology, the incorporation of innovative interventional approaches, such as DCBs or BRSs, holds significant potential for improving clinical outcomes and warrants further investigation within the context of MINOCA management.

Considering the strengths of the present study, it is among the few investigations in the field of MINOCA in which CMR was performed in over 80% of patients, and relatively close to the index event. This approach likely minimized the impact of potential MINOCA mimickers on our conclusions. Moreover, less than 15% of patients were lost during the 2-year follow-up. However, important limitations should be noted. First, CMR was not performed in the entire cohort, and despite the efforts to adhere to the 30-day window, the median time to CMR was 19 days, with a minority of cases delayed up to 144 days. Secondly, our MINOCA population was highly heterogeneous, limiting any conclusions regarding underlying etiological mechanisms, particularly as intravascular imaging and physiology assessments were conducted in only a few cases. The lack of association between initial antithrombotic strategy and long-term outcomes is likely attributable to the relatively small sample size and low event rate. Additionally, treatment adjustments following CMR findings—which altered the initial diagnosis in up to 23% of cases—may have further confounded this relationship. In this regard, a delayed or deferred CMR in a patient with an incorrect initial diagnosis and thus inappropriate therapy might lead to increased risks of bleeding or thrombotic events.

In sum, our investigation unveils the considerable variability in antithrombotic strategies applied to MINOCA patients, reflecting the lack of robust, evidence-based guidance. Relying solely on the working diagnosis of MINOCA and angiographic findings to guide antithrombotic therapy may expose patients to unnecessary bleeding or thrombotic risk. On the contrary, prioritizing cardioprotective therapies and initially adopting low-intensity antithrombotic regimens, with subsequent adjustment based on definitive diagnostic clarification may significantly improve patient outcomes.

## 5. Conclusions

Our study underscores the importance of promptly establishing a definitive diagnosis in patients initially presenting with a working diagnosis of MINOCA. Additionally, determining the optimal antiplatelet therapy remains a major challenge in practice, often guided by coronary angiographic findings. Tailored, evidence-based therapeutic strategies, based on comprehensive patient evaluation and diagnostic precision, play a crucial role in optimizing clinical outcomes. Further clinical trials are needed to draw firm conclusions regarding the optimal medical and interventional management, particularly in cases involving plaque rupture or erosion. In this context, intravascular imaging and CMR emerge as invaluable tools for accurate diagnosis and more precise decision making.

## Figures and Tables

**Table 1:** Baseline characteristics of study population.

	Total (n = 151)	TTS (n = 55)	MINOCA (n = 96)	*p*
Demographics—History				
Age, years	62 ± 12	68 ± 11	59 ± 11	0.065
Female sex, n (%)	107 (71)	50 (91)	57 (59)	<0.001
BMI, kg/m^2^	27 ± 5	26 ± 4	28 ± 5	NS
Smoking, n (%)	94 (43.3)	12 (21.8)	32 (33)	0.001
HTN, n (%)	84 (55.6)	37 (67.3)	47 (49)	<0.001
DM, n (%)	26 (17.2)	8 (14.5)	18 (19)	NS
Dyslipidemia, n (%)	66 (43.7)	28 (51)	38 (39.5)	NS
CKD, n (%)	17 (11.3)	10 (18.2)	7 (7.2)	<0.001
CAD, n (%)	8 (5.3)	3 (5.5)	5 (5.2)	NS
HF, n (%)	1 (0.7)	1 (1.8)	0	NS
AF, n (%)	11 (7.3)	8 (14.5)	3 (3)	<0.001
Family History of CAD, n (%)	20 (13.2)	10 (18.2)	10 (10.4)	0.012
Pre-hospital Medication				
Antiplatelet agents, n (%)	18 (11.9)	6 (10.9)	12 (12.5)	NS
OACs, n (%)	11 (7.3)	7 (12.7)	4 (4)	<0.001
Laboratory Values				
Entry hs-TnI, pg/mL	392 (1–157,000)	425 (3–10,956)	290 (1–157,000)	NS
Peak hs-TnI, pg/mL	715 (25–157,000)	570 (30–10,956)	786 (25–157,000)	0.010
NT pro BNP, pg/mL	919 (50–11,693)	2281 (50–11,693)	255 (70–919)	0.046
Hemoglobin, g/dL	13.4 ± 1.5	13.2 ± 1.3	13.6 ± 1.6	NS
eGFR, mL/min/1.73 m^2^	80 ± 26	74 ± 27	86 ± 26	0.056
InterTAK Score	48 ± 21	61 ± 18	39 ± 19	<0.001
LVEF (%)	51 ± 11	44 ± 12	54 ± 9	0.002
Coronary Findings				
Normal or atherosclerotic vessels, n (%)	95 (63)	41 (74.5)	54 (56)	-
New stenosis < 50%, n (%)	32 (21.2)	11 (20)	21 (22)	-
>50% in non-culprit vessel, n (%)	4 (2.6)	2 (3.6)	2 (2)	-
Epicardial spasm, n (%)	6 (4)	0	6 (6.2)	-
Spontaneous coronary dissection, n (%)	5 (3.3)	0	5 (5.2)	-
Myocardial bridge, n (%)	7 (4.6)	1 (1.8)	6 (6.2)	-

Values are mean ± SD or Median (IQR). Reported comparisons (using Student’s *t*-test and Mann–Whitney U test) were conducted between TTS and all MINOCA patients and *p* values refer to these comparisons. Abbreviations. HTN: Hypertension, BMI: Body Mass Index, DM: Diabetes Mellitus, CKD: Chronic Kidney Disease, CAD: Coronary Artery Disease, HF: Heart Failure, AF: Atrial Fibrillation, OAC: Oral Anticoagulants, hs-TnI: High-Sensitivity Troponin I, NT-proBNP: N-terminal pro b-type Natriuretic Peptide, eGFR: Estimated Glomerular Filtration Rate, LVEF: Left Ventricular Ejection Fraction.

**Table 2:** Multivariate analysis for predictors of antiplatelet intensity (DAPT/DAT).

Predictors	OR	95% CI		*p*
CA findings	0.334	0.307	0.813	<0.001
Age	−0.133	−0.018	−0.001	0.096
ECG	0.057	−0.194	0.434	NS
LVEF	−0.061	−0.190	0.080	ΝS
Prior OAC	−0.224	−1.040	−0.192	0.005

Abbreviations. OR: odds ratio; CI: confidence interval, DAPT: dual antiplatelet therapy, DAT: dual antithrombotic therapy, CA: coronary angiogram; ECG: electrocardiographic abnormalities; LVEF: left ventricular ejection fraction, OAC: oral anticoagulant.

## Data Availability

Data available within text or upon request.

## Abbreviations

The following abbreviations are used in this manuscript:

MINOCA	Myocardial Infarction with Non-Obstructive Coronary Arteries
CMR	Cardiac Magnetic Resonance
LGE	Late Gadolinium Enhancement
AMI	Acute Myocardial Infarction
TTS	Takotsubo Syndrome
DAPT	Dual Antiplatelet Therapy
DAT	Dual Antithrombotic Therapy
SAPT	Single Antiplatelet Therapy
OAC	Oral Anticoagulant
ACS	Acute Coronary Syndrome
RCT	Randomized Clinical Trial
CAD	Coronary Artery Disease
SCAD	Spontaneous Coronary Artery Dissection
OCT	Optical Coherence Tomography
IVUS	Intravascular Ultrasound
CCU	Coronary Care Unit
CRF	Case Report Form
eGFR	Estimated Glomerular Filtration Rate
CRP	C-Reactive Protein
MACEs	Major Adverse Cardiovascular Events
NSTEMI	Non-ST Elevation Myocardial Infarction
STEMI	ST Elevation Myocardial Infarction
UA	Unstable Angina
ICD	Implantable Cardioverter Defibrillator
HF	Heart Failure
AF	Atrial Fibrillation
BMI	Body Mass Index
HTN	Hypertension
DM	Diabetes Mellitus
CKD	Chronic Kidney Disease
NT-proBNP	N-terminal pro b-type Natriuretic Peptide
LVEF	Left Ventricular Ejection Fraction
FFR	Fractional Flow Reserve
PCI	Percutaneous Coronary Intervention
DCB	Drug-Coated Balloon
BRS	Bioresorbable Scaffold
INOCA	Ischemia with Non-Obstructive Coronary Arteries
